# *Teline monspessulana* Can Harm the Chilean Native Tree *Nothofagus obliqua*: Effects on Germination and Initial Growth

**DOI:** 10.3390/plants12193419

**Published:** 2023-09-28

**Authors:** Narciso Aguilera, Lubia M. Guedes, Ulises Alvarado, Katia Sáez-Carrillo

**Affiliations:** 1 Laboratorio de Semioquímica Aplicada, Departamento de Silvicultura, Facultad de Ciencias Forestales, Universidad de Concepción, Casilla 160-C, Concepción 4030000, CP, Chile; lguedes@udec.cl (L.M.G.); ualvarado2016@udec.cl (U.A.); 2 Departamento de Estadística, Facultad de Ciencias Físicas y Matemáticas, Universidad de Concepción, Casilla 160-C, Concepción 4030000, CP, Chile; ksaez@udec.cl

**Keywords:** alkaloids, allelopathy, invasive Fabaceae, phenols, substrates

## Abstract

*Teline monspessulana* is highly invasive in several countries around the world. This species pressurizes and displaces several native and endemic tree species in south-central Chile such as *Nothofagus obliqua*, the native species of greatest timber interest. We determined the effects induced by allelochemical stress of *T. monspessulana* on *N. obliqua* germination and initial growth. Germination was evaluated under in vitro conditions and in natural substrate obtained from sites inhabited by *N. obliqua* and from nearby areas invaded by *T. monspessulana*. Controls irrigated with tap water and treatments with aqueous extracts of aerial organs of the invasive species were used. Morphometric and morphological variables were evaluated, and the composition of alkaloids and phenols from the plant organs used for the aqueous extracts was determined. The substrates were also chemically characterized. Allelochemicals synthesized by *T. monspessulana* caused germination and growth inhibition and tissue-level alterations, as well as leaf and root damage in *N.* obliqua seedlings. In the aerial organs of *T. monspessulana*, the quinolizidine alkaloids aphylline, caulophylline, anagyrine, and sophocarpine were mainly detected. In addition, 21 phenolic compounds were identified, including gallic acid, vanillic acid, chlorogenic acid, p-coumaric acid, and quercetin. The phytotoxic potential of *T. monspessulana* can compromise the natural multiplication of *N. obliqua* and its survival from its first phenological stages. This interdisciplinary study model facilitated the clarification of the plant–plant relationship mediated by allelochemicals. The model can be replicated to investigate other interspecific interactions between invasive and native species.

## 1. Introduction

Several invasive plant species with the capacity to quickly propagate have been described in many ecosystems worldwide [1]. These plants can produce alterations in the community structure of native plants, reduce propagation rates and modify landscape composition, among other impacts [2,3]. Invasive species have developed strategies for the colonization process, such as competition, nutrient cycling disturbance (nitrogen fixation as in Fabaceae/syn Leguminosae) and inducing phytotoxicity [4,5].

These invasion mechanisms are complex, and function simultaneously. Generally, invasive plants cause morphological damage, triggering a cascade of effects which even affect photosynthesis and metabolism [6]. The physiological [7] and morphological evidence [8], as well as oxidative damage [5] induced by allelochemicals in native species, suggest that a focus on the physiological and anatomical basis could shed light on the mechanisms underlying the success of invasive species [9]. Such studies can also predict the impact of invasive species on native populations in invaded ecosystems in the short, medium, and long term [9].

Allelopathy occurs naturally, due to the fact that native species are not adapted to allelochemicals released by exotic species [10]. Accordingly, the selection pressure exerted by biotic and abiotic factors throughout the evolutionary process has promoted the development of numerous biosynthetic pathways in plants, guaranteeing the synthesis and accumulation of secondary metabolites (SMs) at different concentrations [10]. Allelopathic activity is related to distinctive groups of SMs, such as simple phenols, flavonoids, terpenoids, alkaloids, fatty acids, polyacetylenes, sulfuric compounds, oligopeptides and glucosinolates, among others [4]. Phytotoxicity of allelochemicals depends on their bioactive concentration, destination, and persistence in the environment in which they are released [11]. Under natural conditions, allelopathic activity is determined by the synergistic action of several allelochemicals, rather than by the individual action of any specific compound [12]. Allelochemicals can be present in leaves, bark, flowers, fruits, roots, and root exudates [13,14]. Liberation of allelochemicals in the rhizosphere and substrate occurs through leaching of the leaves and other plant aerial organs, release of volatiles, root exudation, and by decomposition of the plant organs deposited on the ground [4,6].

In recent years, allelopathy has been one of the most studied traits of invasive plants [15,16]. Allelopathy has been described frequently in many invasive plant families [17], such as Fabaceae. This plant family has approximately 19,500 described species [18], and at least 27 of them have been reported as invasive in different ecosystems around the world. According to the Center for Invasive Species and Ecosystem Health (https://www.bugwood.org/, accessed on 25 March 2023), Fabaceae is the third group of plant families with the largest number of invasive species. The presence of some allelochemicals (e.g., quinolizidine alkaloids and nonstructural amino acids) have been detected in many species of the subfamilies Mimosoideae and Papilionoideae (or Faboideae) [19,20].

Several highly invasive Fabaceae species are reported in Chile, constituting a threat to the different forest ecosystems [21]. One of these species is *Teline monspessulana* (L.) K. Koch (Papilionoideae), which is native to the Mediterranean Basin [22]. This species is synonymized as *Genista monspessulana* (L.) L.A.S. Johnson, and in South American countries, where it is reported as invasive, it is known as “retama”, “retamilla”, and “retamo liso”, but in other languages it is named French, Cape and Montpellier broom. In Chilean conditions, this species forms dense populations that eliminate the native vegetation, affect local fauna, interrupt water flows, alter soil nutrient cycles and increase the risk of propagation and intensity of forest fires [21]. The presence of quinolizidine alkaloids, flavonoids and isoflavones has been reported in aerial organs of *T. monspessulana* [23,24,25], but to our knowledge there are no previous studies of the allelopathic effect of the compounds released by this species. For both phenols and alkaloids, an allelopathic effect has been reported associated with the inhibition of germination, rooting and growth of other plants, the reduction in biomass, and chlorophyll accumulation [26]. The detection of quinolizidine alkaloids and phenols in aerial organs of *Acacia dealbata* Link (Fabaceae) have been related to the inhibitory effect of germination and early growth of the Chilean native species *Quillaja saponaria* Molina (Quillajaceae) [27] and eight native Spanish species [7].

The biodiversity of the Chilean native forest forms a unique, natural world heritage, and its south-central region is considered one of the 35 global biodiversity hotspots [28]. However, deforestation, fragmentation and change of land use are a latent risk for native trees, particularly species of Notofhagaceae [29,30]. *Nothofagus obliqua* (Mirb.) Oerst. is one of those species, which is exposed to the advance of the *T. monspessulana* colonization front and its allelopathic potential. In field observations, little or no presence of *N. obliqua* seedlings near the areas invaded by *T. monspessulana* has been observed. *Nothofagus obliqua*, known as “roble”, has great commercial importance. In addition to its ornamental value, *N. obliqua* produces good-quality wood and firewood, representing the main source of wood among Chilean native angiosperms [31]. In this context, it is hypothesized that the continuous exposure of the native species *N. obliqua* seeds and seedlings to the allelochemicals released by the invasive *T. monspessulana*, decreases their germination potential and initial growth, causing morphoanatomical alterations that compromise the seedling survival. To corroborate this hypothesis, the chemical profile (phenols and alkaloids) of the aerial organs of *T. monspessulana* was characterized, and the effect of this extract on the germination, initial growth and morphoanatomy of *N. obliqua* seedlings was also evaluated.

## 2. Results

### 2.1. In Vitro Assay: Morphometric Measurements

**Figure 1:**
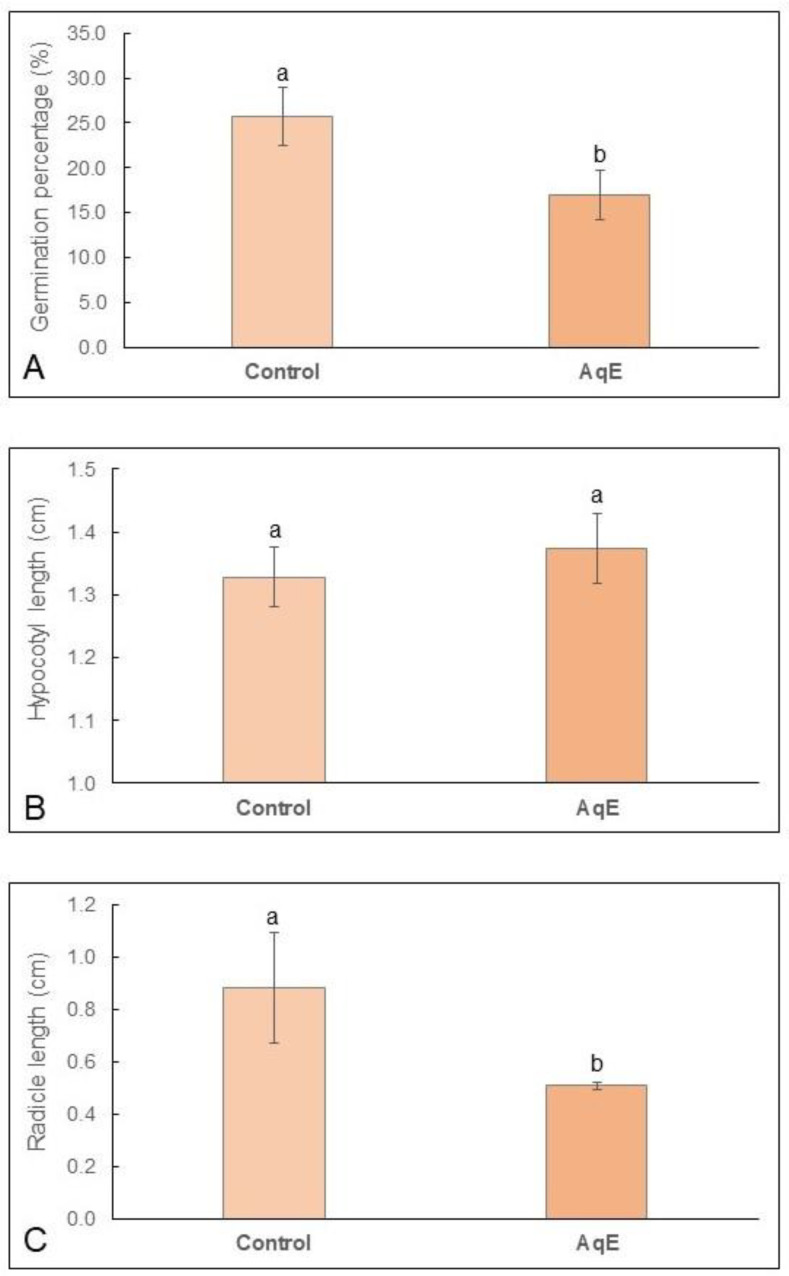
Effects induced by aqueous extracts of *Teline mospessulana* (AqE) on germination (**A**), radicle length (**B**), and hypocotyl length (**C**) of *Nothofagus obliqua* seedlings in in vitro assays. Control seedlings grown in water. Data in B and C were represented by the mean and standard deviation. A *p* value of <0.05 indicate significant differences between treatments after the Student’s *t* test. In the control and treatment, data were obtained from the analysis of 10 Petri dishes (n = 10). Different letters mean significant differences.

**Figure 2:**
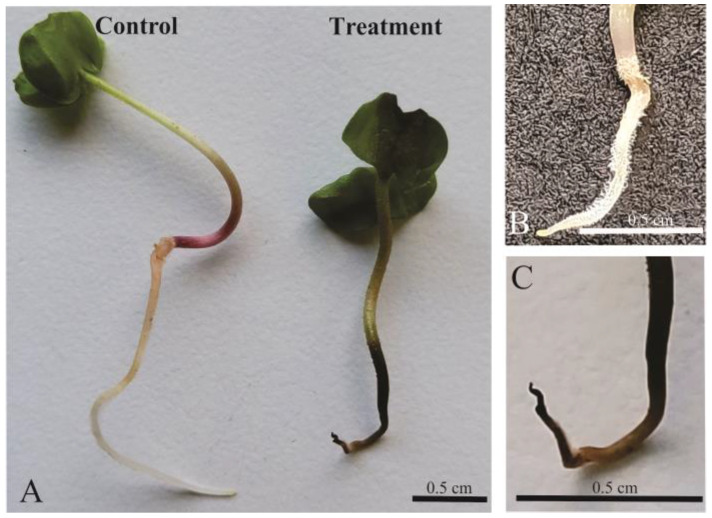
Morphology of *Nothofagus obliqua* seedlings from in vitro assay. (**A**) Comparison of seedlings germinated in water (control) and in *Teline monspessulana* aqueous extract (treatment). Note the difference between the color and vigor of the radicle of the control seedlings (**B**) and the necrosis induced by the aqueous extract (**C**). Pictures were taken by the principal researcher.

*Nothofagus obliqua* seeds had a low germination percentage (25.7%), which decreased significantly (*p* = 0.004) under the effect of the aqueous extract of *T. monspessulana* (16.94%) (Figure 1A). This extract produced a significant decrease in radicle length (*p* = 0.007) (Figure 1B and Figure 2A) and induced root tip necrosis (Figure 2A,C), but did not affect the hypocotyl length (*p* = 0.820) (Figure 1C). At 20 days of evaluation, the radicle of the control seedlings had an average length of 0.88 cm (Figure 1A) and had a white coloration with a long piliferous zone (Figure 2A,B). The radicle length of the seedlings that sprouted under the influence of aqueous extract reached 0.50 cm in length (Figure 1A), were dark brown, and the piliferous zone was not differentiated (Figure 2C).

### 2.2. Assay in Substrate

#### 2.2.1. Dynamics of Germination and Morphometric Variables

**Figure 3:**
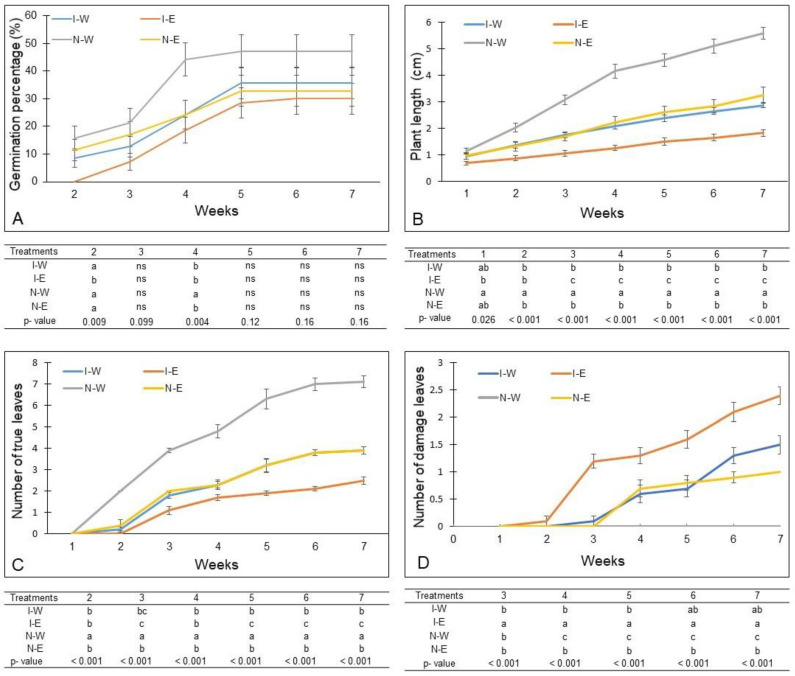
Growth dynamics of *Nothofagus obliqua* seedlings that grew for seven weeks in four treatments: native substrate + water (N-W), native substrate + aqueous extract (N-E), invaded substrate + water (I-W), invaded substrate + aqueous extract (I-E). (**A**) Germination percentage, (**B**) Plant length, (**C**) Number of true leaves, (**D**) Number of damaged leaves. The table below each graph indicates the differences between treatments for *p* ≤ 0.05 after a one-way ANOVA and Tukey’s Test for post hoc comparison. Different letters between the treatments in each evaluation week mean significant differences. Each variable was evaluated weekly in 10 plants from each treatment and are represented by their mean and standard deviation.

*Nothofagus obliqua* seeds began to germinate at two weeks, except in I-E treatment. The highest germination occurred between the third and fourth week in N-W, reaching around 45%, with a minimum increase until the seventh week (Figure 3A). This treatment exhibited a significantly higher germination percentage than the other three treatments (Figure 3A). The I-W, I-E and N-E treatments maintained a relatively similar behavior as a function of time. Only during the second and fourth week were there significant differences between the treatments. In the second week of treatment, for I-E there was no germination, differing significantly from the other treatments (*p* = 0.009), while in the fourth week, the N-W treatment had a significant increase in the number of germinated seeds in comparison to I-W, I-E and N-E treatments (*p* = 0.004) (Figure 3A). These treatments (I-W, I-E and N-E) reached the highest germination percentage (30–35%) in the fifth week and this remained so until the end of the experiment. During the seven weeks of evaluation, treatment I-E maintained the lowest germination percentage.

**Figure 4:**
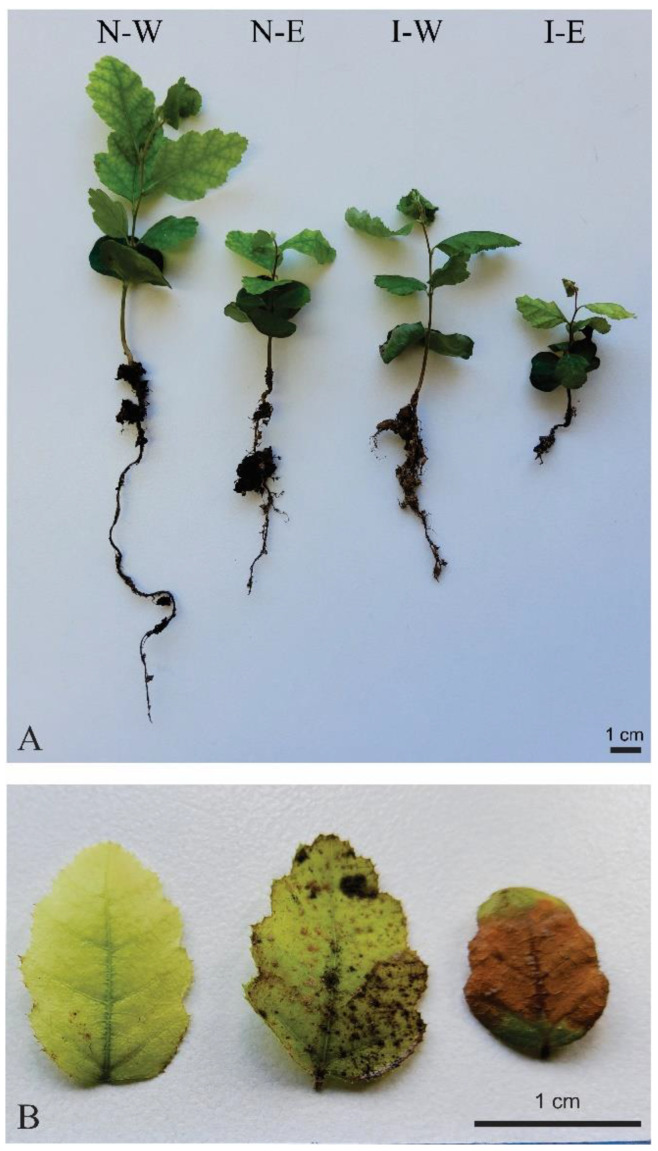
*Nothofagus obliqua* seedlings grown in four different treatments: native substrate + water (N-W), native substrate + aqueous extract (N-E), invaded substrate + water (I-W), invaded substrate + aqueous extract (I-E). (**A**) Morphological aspect of seedlings. Note signs of dehydration in I-W and I-E treatments, more intense in the last treatment. (**B**) Different degrees of leaf damage observed in I-W and I-E treatments.

*Nothofagus obliqua* seedlings grew progressively during the experimental period, but with significant differences between treatments. During the experimental period (seven weeks) the seedlings in N-W reached a significantly larger size with respect to the other treatments (Figure 3B and Figure 4). In the second week, the LP did not show significant differences between N-E, I-W and I-E (Figure 3B). Although the seedlings in N-E and I-W grew significantly less than in N-W, no significant differences were observed between either treatment (Figure 3B). However, from the third week, the growth of the *N. obliqua* seedlings in I-E was significantly less than in the other three treatments, barely exceeding 1.8 cm in length in the seventh week (Figure 3B). Over time, the seedlings of the N-W treatment grew two times more than in the N-E and I-W and elongated approximately five times more than those grown in the I-E.

From the second week, the *N. obliqua* seedlings in the N-W treatment developed a significantly higher number of leaves with respect to the other treatments, increasing progressively as the weeks progressed (Figure 3C). The N-E and I-W treatments did not show significant differences in the numbers of true leaves, and both treatments developed a significantly higher number of leaves than for the N-E treatment (Figure 3C). Both N-E and I-W treatments inhibited the formation of true leaves by more than 50%, and I-E caused approximately 70% inhibition with respect to the control, maintaining this trend during the seven weeks (Figure 3C). The combination of *T. monspessulana* extract and invaded substrate (I-E) induced greater leaf damage than the other treatments, and this was visible from the second week, increasing until the seventh week. At the end of the seventh week, leaves were damaged with chlorosis and/or necrosis (Figure 4A,B). Some type of foliar damage was induced in the I-W treatment from the third week and in the N-E from the fourth week (Figure 4B), but without significant differences between them (Figure 3D). From the sixth week, the number of damaged leaves in the I-W treatment did not show significant differences to the I-E treatment (Figure 3D).

The interactions between substrate types (native or invaded) and the irrigation component (water or aqueous extracts) did not show significant differences for the morphometric variables of *N*. *obliqua*. However, when comparing the native substrate with the invaded substrate, a significant decrease in plant growth was noted in the invaded substrate (Table 1, Figure 4A). Similarly, in the invaded substrate, *N*. *obliqua* seedlings formed significantly fewer leaves and the main root length was significantly reduced compared to the native substrate (Table 1, Figure 4A). Seedlings of *N*. *obliqua* irrigated with water exhibited significantly higher values in their growth, formation of true leaves and length of the main root (Table 1, Figure 4A). The opposite result was recorded when *N*. *obliqua* seedlings were irrigated with aqueous extract of *T*. *monspessulana* (Table 1, Figure 4A).

#### 2.2.2. Anatomical Analysis

**Figure 5:**
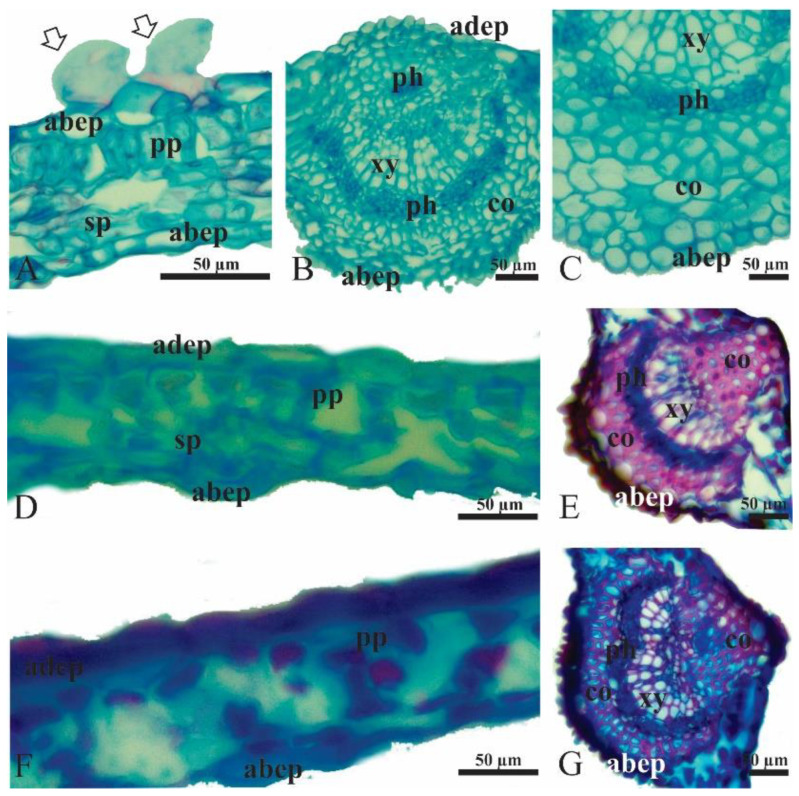
Anatomical features of *Nothofagus obliqua* seedling leaves grown under different treatments. Leaves from seedlings grown in: (**A**–**C**) native substrate and irrigated with water (N-W); (**D**,**E**) native substrate and irrigated with *Teline monspessulana* extract (N-E); (**F**,**G**) invaded substrate and irrigated with water (I-W). (**A**,**D**,**F**) Mesophyll. White arrows in (**A**) point to two glandular trichomes. (**B**,**C**,**E**,**G**). Midrib. Note in (**D**,**F**) the disorganization of the mesophyll and symptoms of dehydration as a result of the treatments. Note in (**B**,**C**) the absence of lignified cell walls (no red staining); in (**E**,**G**) the lignification of the midrib (red staining) is denoted as a consequence of the treatments. Abbreviations: abep: abaxial epidermis, adep: adaxial epidermis, co: collenchyma, ph: phloem, pp: palisade parenchyma, sp: spongy parenchyma, xy: xylem. All scale bars indicate 50 μm.

*Nothofagus obliqua* leaves have a dorsiventral mesophyll, with a palisade parenchyma layer towards the adaxial face and two layers of spongy parenchyma towards the abaxial face (Figure 5A). Both abaxial and adaxial epidermis are uniseriate (Figure 5A). Ordinary epidermal cells, in the transversal section, are more globose on the adaxial side and more elongated on the abaxial side (Figure 5A). Glandular trichomes form near the leaf edges (Figure 5A). The vascular system consists of a prominent major vein and numerous minor veins. Each vein contains both xylem and phloem tissues. The midrib is formed by a bicollateral vascular bundle, surrounded by layers of collenchyma, forming a prominent rib towards the abaxial face (Figure 5B,C). The extract of *T. monspessulana*, regardless of the substrate where the *N. obliqua* seedling grew, induced leaf structure disorganization (Figure 5D,F). The ordinary cells of both the adaxial and abaxial epidermis collapsed, and no trichomes were dedifferentiated (Figure 5D,F). The palisade parenchyma cells lost their tubular shape, and the spongy tissue was scarce, with large intercellular spaces (Figure 5D,F). Midrib abaxial and adaxial collenchyma cells showed strongly lignified cell walls (Figure 5E,G).

#### 2.2.3. Chemical Characteristics of the Substrates

Substrates were mainly the product of the decomposition of leaf litter and other plant materials from *N. obliqua* (native substrate) and *T. monspessulana* (invaded substrate). Both substrates had some relatively similar chemical components, but their biggest difference consisted in the phosphorus, exchangeable potassium, and available potassium contents (Table 2). Although the available nitrogen presented high values in the two substrates, the native substrate showed concentrations of approximately 27% more nitrogen available to the plants (Table 2).

#### 2.2.4. Alkaloid Profile of *T. monspessulana*

The only alkaloid detected in the four organs of *T. monspessulana* (leaves, stems, flowers and pods) was aphylline, although in greater quantity in stems and flowers (Table 3, Figures S1–S4). Aphylline was the only alkaloid identified in stems, while in pods anagyrine was also detected. In contrast, five and seven alkaloids were detected in the flowers and leaves, respectively. In both plant organs, caulophylline, anagyrine, and sophocarpine were found (Table 3). Additionally, lupanine was identified in flowers, and psilocin, ellipticine and cytisine were found in leaves. The abundance of these compounds was variable in the different plant organs, ranging from small to considerable relative amounts, expressed as relative peak area to total peak area per plant organ (Table 3).

#### 2.2.5. Phenol Profile of *T. monspessulana*

In the *T. monspessulana* leaves, stems, flowers, and pods, 21 phenolic compounds were detected, 15 of them in leaves and flowers, 13 in bark, and 4 in pods (Table 4). The most abundant compounds per organ were vanillin and quercetin 3-glucoside in leaves, quercetin 3-glucoside and p-coumaric acid in flowers, 14-hydro-ybenzoic and 3,4-dimetho-ybenzyl alcohol in bark, and vanillic acid and p-coumaric acid in pods (Table 4). Four similar compounds were detected in the four organs, although with significantly different concentrations (Table 4). Concentrations of the compounds were highly variable among organs. For example, the concentration of 14-hydroxy benzoic acid was 8.7 and 14.8 times higher in bark than in flowers and leaves, respectively (Table 4). Similarly, the concentration of gallic acid was significantly lower in flowers (3.4 times), leaves (5.3 times), and pods (3.1 times) than in bark (Table 4). The concentrations of vanillic and caffeic acids and quercetin were significantly higher in stems than in flowers and leaves (Table 4). On the other hand, flowers had significantly higher concentrations of vanillin, quercetin 3-glucoside, chlorogenic and p-coumaric acids, compared to other organs. The p-coumaric acid decreased significantly in leaves (41 times), bark (26.8 times), and pods (6.31 times) compared to flowers (Table 4). Kaempferol was the only phenolic compound detected in significantly higher concentrations in the leaves than in the flowers and stems (Table 4). However, there were not only differences between the four organs in concentrations but also in composition, with the detection of nine compounds that are only present in some organs. For example, in the stems, the unique presence of 3,4-dimethoxy benzyl alcohol, acid 5-(hydroxyl methyl)furfural and p-tyrosol was detected, and in the leaves myricetin, acid apigenin and catechin were detected, while in flowers ellagic acid, epicatechin and pinocembrin were detected, which were not detected in the other organs (Table 4).

## 3. Discussion

The percentages of natural germination of *N. obliqua* are naturally low, ranging from approximately 15 to 25% [33], and this is in line with our results. Under both in vitro and substrate conditions, the *N. obliqua* germination was affected by *T. monspessulana*, which can limit the number of individuals that can multiply from the reduced seed banks of this native species. The seeds and flowers of *N. obliqua* are consumed by burrowing parakeets (*Cyanoliseus patagonus* Vieillot) in some areas [34], which further decreases the seed availability for the multiplication of new plants. In these areas, the effects of *T. monspessulana* on *N. obliqua* may be even more dangerous.

The allelochemicals present in aqueous extracts of *T. monspessulana* interfered with the germinative process from the first weeks in the two substrates. However, when these allelochemicals were deposited and transformed in the invaded soil, the effect on germination was greater. Allelochemicals in the soil can act directly on other plants, or indirectly, due to degradation or transformation by soil microorganisms, interference with the development of surrounding plants, or changes in the soils’ abiotic factors [35]. The incorporation of foliage from invasive Fabaceae such as *Cytisus scoparius* (L.) Link and *Ulex europaeus* L. in the soil reduces the density and size of weeds [36]. A similar result was observed when soybean seeds (*Glycine max* (L.) Merrill) were exposed to substrates enriched by plant remains of *Avena strigosa* Schreb, *Raphanus sativus* L., *Vicia sativa* L. and *Lolium multiflorum* Lam. [37]. It can be reasoned that allelochemicals remain in leaf litter and plant remains and are released as organic matter decomposes. These compounds in the soil could affect the ability of other plants to take up or modify the mobility of nutrients through processes such as biological inhibition of nitrification [38], which could in turn contribute to explaining the chlorosis in plants subjected to the treatments.

*Nothofagus obliqua* seedlings that remained under allelochemical stress caused by *T. monspessulana* (substrate and extract) showed increasing symptoms of premature aging and deterioration. In addition to an incipient irregular defoliation in several seedlings, chlorophyll degradation was noted in the treatments, evidenced by the progressive appearance of chlorosis, until leaf necrosis. There are not many studies on the effects of allelopathic stress on leaf tissue. However, the present results indicated that at a morphological level, the number of leaves with leaf damage increased considerably in the seedlings that grew in the invaded soil, which was even higher in seedlings irrigated with the extract. At the anatomical level, disorganization of the mesophyll and lignification of the midrib were observed. On the mesophyll, gas exchange occurs from the substomatal cavity to the carboxylation sites [39,40]. Mesophyll conductance has an important impact on photosynthesis and it is estimated that mesophyll structural changes can limit the leaf photosynthetic activity [39,40]. The mesophyll disorganization of *N. obliqua* leaves irrigated with aqueous extracts can also alter photosynthesis and gas exchange and, therefore, inhibit the growth of the seedlings.

At the morphological level, the disruption in photosynthesis was reflected in leaf chlorosis, mainly in treatments with invaded soil. Allelochemicals can cause water stress by inhibiting the activity of ions (e.g., Na^+^, K^+^) and by inducing the production of reactive oxygen species, as well as altering the activity of antioxidant enzymes. Plant responses to allelochemicals can be similar to biotic and abiotic stress responses [41]. This may explain the dehydration of *N. obliqua* seedlings, especially in the treatment of the invaded substrate irrigated with aqueous extract of *T. monspessulana*, and the lignification of xylem cells and vascular collenchyma. Lignin guarantees resistance to chemical attacks, and its polymerization depends on the formation of oxidative enzymes, mainly the peroxidase enzyme [42]. Early lignification in roots and leaves of seedlings under allelochemical stress could limit the root growth and expansion of target seedlings such as *N. obliqua*. Structural damage in seedlings under allelochemical stress has been reported in *A. dealbata* roots, where the formation of root hairs was inhibited, the epidermis and parenchymal tissue were destroyed, and the vascular system collapsed [43]. In an auto-allelopathic experiment, also with *A. dealbata*, the same behavior at root level was observed [16].

Both the invaded substrate and *T. monspessulana* extract affected plant and root length and leaf number formed, indicating that *T. monspessulana* interferes with the initial growth of *N. obliqua*. Previous work indicates that *N. obliqua* is sensitive to the allelopathic effects induced by extracts from aerial parts of *A. dealbata*, another invasive Fabaceae from south-central Chile [27]. The growth retardation of *N. obliqua* seedlings is probably related to the ability of some allelochemicals to interrupt metabolic processes, especially the concentration of hormones [6]. A notable effect of allelochemical stress is the inhibition of root growth or lateral root proliferation, probably due to the accumulation of auxin in the roots [44]. Similarly, a smaller number of leaves and leaf damage means lower photosynthetic capacity, an effect described in allelopathic processes [45]. The effect of the allelochemicals of *T. monspessulana* on the root system of *N. obliqua* implies deficiencies in the uptake of nutrients from the soil, while a lower photosynthetic capacity decreases the availability of nutrients, mostly carbon sources, for plant growth.

The substrates had relatively different compositions, due to their origins. The higher content of N, P and K available in the substrate formed under the *N. obliqua* canopy could have favored the initial growth of this native species. The effect of these macronutrients on the growth and development of plants is widely recognized [46]. However, when the substrate receives a systematic deposition of *T. monspessulana* allelochemicals, the growth of *N. obliqua* seedlings can also be affected. As the colonization front of the plant invader approaches the native species, the substrate that the native species inhabits progressively receives plant and allelochemical remains of *T. monspessulana*. Over time, such a substrate could transform and resemble the substrate under the *T. monspessulana* foliage. This scenario would directly affect the germination and natural regeneration of *N. obliqua*, as demonstrated in this study. As *T. monspessulana* penetrates the remnants of native tree populations, the substrate will gradually change its chemical composition, making it unfavorable for seedling growth.

Most of the chemical compounds produced by Fabaceae species have high biological activity, related fundamentally to quinolizidine alkaloids and phenols [19,47,48]. For both groups of secondary metabolites, an allelopathic effect has been reported which is associated with the inhibition of germination, rooting and growth of other plants, biomass reduction, and decrease in chlorophyll content [26]. Alkaloids can be interspersed in DNA and affect enzymatic activity. Quinolizidine alkaloids produced by Fabaceae could especially alter membrane permeability and protein synthesis, affecting the function of some tissues or leading to cell death [49].

This investigation revealed that the aerial organs of *T. monspessulana* (flowers, leaves, stems, and pods) are composed of several quinolizidine alkaloids. Depending on alkaloid type, they were distributed in a coincident or divergent way in the different aerial organs. Quinolizidine alkaloids are recognized for their phytotoxic activity. For example, aphylline is abundant in *Lupinus* spp. Due to the high toxicity associated with aphylline and other quinolizidine alkaloids, some *Lupinus* spp., such as *L. montanus* HBK, *L. stipulatus* Agardh and *L. aschenbornii* Schauer cannot be used as protein sources [50]. Aphylline was the alkaloid detected in the highest amount in all *T. monspessulana* organs.

Similarly, anagyrine has insecticidal and cytotoxic activity [51] and has a potent teratogenic activity for cattle [52]. Caulophylline is also recognized as highly bioactive, with use in medicine [53]. Sophocarpine has been used to try to control the red imported fire ant (*Solenopsis invicta* Buren) [54]. Lupanine and cytisine exert great antifungal activity against the mycelia of *Fusarium oxysporum* Schltdl. [55], and it is suggested that they have high toxicity and teratogenic activity [56]. Lupanine, also detected on *T. monspessulana* flowers, has an inhibitory effect on the germination of some plant species [57]. Ellipticine was first isolated from *Ochrosia elliptica* Labill and has potent anticancer properties. It has several modes of action, one of the most recognized being DNA intercalation and topoisomerase II inhibition [58]. As far as we know, there are no reports of previous allelopathic studies on plant–plant interaction regarding *T. monspessulana*. However, a study reported some of these alkaloids to be present in *T. monspessulana* [59], and they have been reported for other Fabaceae species [60].

Phenolic compounds, with recognized biological activity [61,62,63], were also identified in the aerial organs of *T. mospessulana*. Gallic and chlorogenic acids, detected in flowers, stems, leaves and pods, have herbicidal activity [64]; the latter has nematicidal action [65]. Similarly, p-coumaric acid is phytotoxic against *Lepidum sativum* L., *Lactuca sativa* L. and other herbaceous species, and is autotoxic for *Asparagus officinalis* L. [66]. Finally, in leaf litter of different decomposition times from different species, such as *Ailanthus altissima* [Mill.] Swingle, *Robinia pseudoacacia* L., *Ulmus pumila* L., *Populus alba* L., *Populus nigra* L. and *Ulmus minor* Mill., quercetin and vanillic acid have been identified [67], inhibiting the growth of understory species.

Polyphenols and alkaloids composed the aqueous extract with which *N. obliqua* was irrigated in the treatments. These compounds are gradually released and incorporated into the invaded substrate. Microorganisms from the soil or substrate may transform the compounds, enhancing or inhibiting their biological activity [9]. However, donor plants constantly synthesize and release these compounds [68], turning allelochemical stress into a chronic process [27,69].

## 4. Conclusions

Our research contributes to the clarification of the plant–plant relationship mediated by allelochemicals, and in this context reports some specific novelties. For the first time it is revealed that invasive *T. monspessulana* has a high allelopathic potential based on various phytotoxic compounds, such as quinolizidine alkaloids and phenolic compounds, supported by in vitro and substrate assays. It is also the first time that the biotic relationship between this invasive species and the important native tree species *N. obliqua* has been studied from a semiochemical perspective. *Teline monspessulana* reduces germination and interferes with the initial growth of *N. obliqua*, causing considerable morphological and anatomical damage. The integration of morphoanatomical and chemical studies of the substrates and of *T. monspessulana* allowed us to better understand the cause–effect relationship between the invasive donor species and the native recipient species, in this case *N. obliqua*. Chronic allelochemical stress resulting from the constant release of highly bioactive compounds from *T. monspessulana* caused phenotypic changes defined by lower growth of *N. obliqua* seedlings and increasing foliar deterioration, until defoliation. This is a behavior representative of premature aging in seedlings, supported by underlying lignification and increasing tissue alteration in leaves and roots. Our results suggest that the constant exposure of *N. obliqua* to allelochemical stress induced by *T. monspessulana* may constitute a risk for the natural multiplication of this native species—of timber interest—in the forest remnants of south-central Chile. The survival of *N. obliqua* seedlings established in areas close to the *T. monspessulana* colonization front may be compromised. This would explain the absence of *N. obliqua* seedlings in the vicinity of the *T. monspessulana* monocultures recorded in field observations. This study model can be applied to other native tree species that cohabit in the same distribution range as *T. monspessulana* in south-central Chile or in other ecosystems, in which this invasive species represents a threat to native plants.

## 5. Materials and Methods

### 5.1. Sampling Sites and Plant Material

Samples of *T. monspessulana* were collected on a hill located on campus at the University of Concepción (UdeC, Spanish acronym) (36°50′09.4″ S 73°01′49.9″ W), Biobío Region, Chile. The invasive *T. monspessulana* and *Acacia dealbata* Link (Fabaceae) are dominant in the sampling area, forming large patches of monoculture and with spaces alternating between both invasive Fabaceae species. Isolated native trees of *N. obliqua*, *Q. saponaria*, *Peumus boldus* Mol. (Monimiaceae) and *Cryptocarya alba* Mol. (Lauraceae) are also frequent. The colonization front of the invasive species is rapidly approaching the area inhabited by the native trees. Flowers, leaves, stems and pods were collected from the invasive plant. Reproductive organs were collected between September and December 2020, and vegetative organs between January and June 2021. *Nothofagus obliqua* seeds were collected from specimens located on the UdeC Campus. Plant materials were wrapped in kraft paper, placed inside plastic bags, and transferred to the Applied Semiochemical Laboratory (LSqA, Spanish acronym) (http://lsqa.udec.cl/, accessed on 25 March 2023) for processing.

### 5.2. Preparation of Aqueous Extract

*Teline monspessulana* aerial organs were macerated in distilled water (250 g L^−1^) in an Erlenmeyer flask in the dark at room temperature for 4 days [69]. During the first 48 h, the Erlenmeyer flasks were placed in a mechanical shaker at 210 rpm (DLAB SK-L330-Pro, China). Subsequently, the liquid was filtered through Whatman 1 filter paper and 2 mL L^−1^ of PPM (plant preservation mixture) was added. The PPM prevents proliferation of microorganisms in the aqueous extract, mainly fungi.

### 5.3. In Vitro Bioassays

Bioassays were performed in glass Petri dishes with a diameter of 9 cm. A Whatman No. 1 filter paper disk was placed on each dish, 3 mL of aqueous extract was added, and 23 *N*. *obliqua* seeds were distributed homogeneously on the filter paper. A control was established with 3 mL of distilled water and PPM (2 mL L^−1^). The Petri dishes, control and treatments (n = 10) were covered and sealed with Parafilm^®^ to avoid desiccation. They were placed in a growth chamber at a temperature of 20 ± 2 °C, relative humidity of 60 ± 5%, light intensity of 50 μmol m^−2^ s^−1^ and a photoperiod of 16/8 h (light/dark). However, the Petri dishes were kept at low light (5 μmol m^−2^ s^−1^) until germination started. The bioassay lasted 20 days and was reviewed daily. Evaluations of the morphometric variables followed the methodology describe by Aguilera et al. [69]. The germination percentage (PG), radicle (LR) and hypocotyl length (HL) were evaluated at the end of the experiment.

### 5.4. Bioassays in Substrate

#### 5.4.1. Substrate Collection and Preparation

Substrate was collected under *T. monspessulana* foliage (invaded substrate) and under the canopy of the native *N*. *obliqua* (native substrate). These substrates were collected in five sampling sites containing both tree species. The sites were located at 0.20-to-1.0 m from the stems, without exceeding the drip area. Leaf litter and other plant debris deposited on the ground (A0 horizon) were removed. The substrate was placed in nylon Ziploc bags and was sieved to remove coarse particles and stones. All substrate sample fractions were gathered in a pool and used in the subsequent assays.

#### 5.4.2. Bioassay Establishment

Bioassays were established in plastic trays for seedlings of 50 cells (50 × 46 × 30 mm; 0.073 l per cell). Each cell was filled with substrate and two *N. obliqua* seeds were sown (n = 100 per treatments). Four treatments were established: (i) native substrate + water (N-W), (ii) native substrate + aqueous extract (N-E), (iii) invaded substrate + water (I-W) and (iv) invaded substrate + aqueous extract (I-E). Trays were covered with a transparent lid and transferred to the growth chamber under the conditions described above. A moistening irrigation with water was applied for 3 days to all treatments. Subsequently, watering was started every 3 to 4 days with water or aqueous extract, according to the treatment. The experiments were inspected daily to verify the humidity status and eliminate weed shoots. Every 7 days for 7 weeks, the following morphometric variables were evaluated: number of germinated seeds, plant length (PL), number of true leaves (NTL), and number of damaged leaves (NDL). After 49 days, the length of main root (LMR) was also evaluated.

#### 5.4.3. Anatomical Analysis

The third and fourth leaves of the *N. obliqua* seedlings from each treatment (n = 5) were collected. Leaves were counted from the base towards the stem apex. In the I-E treatment, leaves were dehydrated (Figure 4) and could not be collected for the anatomical study. The leaves were cut in the middle region into 1 cm^2^ segments. The leaf segments were fixed in FAA (formalin, acetic acid, and 70% ethanol; 1:1:18, *v*/*v*/*v*,) [70] for 48 h. The samples were subsequently dehydrated in a series of butyl alcohol (70, 80, 90 and 100%) and included in Paraplast Plus^®^ (Sigma-Aldrich, Darmstadt, Germany) [71]. The samples were sectioned in a rotatory microtome (Leica RM2125RTS, Leica Byosistems, Nussloch, Germany) between 10 and 12 µm, and affixed on slides with Bissing’s adhesive [72] to a hot plate, at 40 °C. After 24 h, the sections were deparaffinized in butyl acetate, hydrated in ethanolic series (100, 90, 80, 70 and 50%) [71] and stained with Astra blue-safranin (9:1 *v*/*v*; [73], modified to 0.5%). All samples were dehydrated in ethanol series and mounted with Pertex^®^ embedding medium. All samples were analyzed and photographed using a photomicroscope (Leica DM500, Leica Byosistems, Nussloch, Germany) coupled to a digital camera (Leica ICC50, Leica Byosistems, Nussloch, Germany).

### 5.5. Chemical Substrate Analysis

Substrate samples (native and invaded) were air-dried and homogenized for chemical measurements. Substrate pH was determined in a 1:2.5 substrate-to-solution ratio in water and CaCl_2_ (0.01 M) [32]. Substrate N and C contents were determined by loss of ignition via a CHN IRMS Analyzer (Sercon Ltda, Santiago de Chile, Chile). Phosphorus availability was determined using the Olsen method [74]. The cation potassium (K^+^) was measured using an atomic absorption and emission AAS Spectrophotometer (A Analyst 400, PerkinElmer) [32]. Electrical conductivity and organic matter were also determined according to the methodology described by Sadzawka et al. [32]. All analyses were carried out in the Soil, Plant and Water Laboratory of the Faculty of Agronomy, University of Concepcion. The establishment of content levels of the substrate elements was carried out according to the ranges established by Sadzawka et al. [32].

### 5.6. Characterization of the Alkaloid Profile

Equal amounts (100 g) of leaves, stems, flowers, and pods of *T. monspessulana* were weighed and placed separately in methanol extraction (1:4, *w*/*v*). Each extract was macerated at room temperature in darkness. After seven days, the methanol extracts were filtered and dried under reduced pressure in a rotary evaporator (LabTech, Sorisole, Italy) coupled to an empty pump (v-700-Buchi, Flawil, Switzerland), at 40 °C. The dry extracts of leaves, stems, pods, and flowers were resuspended in a 10% hydrochloric acid aqueous solution (10 mg of dry extract per ml of aqueous HCl) [75]. After 2 h, the extracts were sonicated (Elmasonic S 30 H, Singen, Germany) and filtered. The acidic extracts were basified with 10 M sodium hydroxide solution to pH 9. The basified extracts were extracted in a funnel with chloroform (4 times). Organic fractions were collected and pooled. Both the organic and aqueous fractions were monitored using thin-layer chromatography (TLC) in a dichloromethane: methanol (8:2) mobile phase to corroborate the presence/absence of alkaloids. The chloroform fractions were dried in a vacuum at 37 °C and used for alkaloid identification.

An aliquot (20 mg) of each chloroform fraction (leaves, stems, pods, and flower fractions) were resuspended in 300 µL of ethyl acetate for alkaloid identification using gas chromatography coupled with mass spectrophotometry (GC-MS) in an Agilent gas chromatograph (7890A) with a splitless injector (250 °C) and Agilent mass detector (5975C), using a capillary column of fused silica type HP5-MS, 30 m, 0.25 mm internal diameter, and 0.25 mm thick, under the following characteristics: temperature: 250 °C; detector (mass): 280 °C; oven: initial 100 °C for 5 min, increasing to 8 °C/min up to 250 °C and maintained for 15 min. The adjustment of the detector as a scanner varied from 50 to 500 amu. The flow of carrier gas (electronic grade helium) was set at 1 mL min^−1^ [76]. Tentative identification of the alkaloids was made by matching the mass spectra with the records in NIST 17 (NIST/EPA/NIH MASS Spectral Library 2017) and comparing the spectra obtained with those reported in the literature. NIST 17 collects representative alkaloids from all referenced skeletons. The structure of an alkaloid was tentatively assigned when the overlap with the database exceeded a 90% match. The skeletons of unidentified compounds were proposed according to the index of greatest similarity with those that appear in the database. The percentage of compounds in the extracts was calculated based on the total area of the GC-MS peaks [77].

### 5.7. Characterization of Phenol Profiles

Polyphenol extraction was performed according to the methodology described by Soto et al. [78]. Leaves, stems, flowers, and pods of *T. monspessulana* were freeze-dried and ground separately. From each ground sample, 0.5 g was macerated in 10 mL of hydroalcoholic solution (50% distilled water: 50% methanol) at 50 °C for 16 h. Extracts were filtered through Whatman N°1 filter paper and stored at 4 °C for polyphenol quantification. For each sample (leaves, stems, pods, and flowers), 10 µL of hydroalcoholic extract (100 µg mL^−1^) was used for identification and quantification of phenols using high-performance liquid chromatography with a diode array detector (HPLC-DAD, Hitachi Primaide, Tokyo, Japan). The HPLC was equipped with a column Kromasil^®^ C18 and separation was performed with a mobile phase of 1% formic acid in water (A) and acetonitrile (B) at constant solvent flow of 1 mL min^−1^. The detector was set at 250, 280, 320, and 360 nm. To determine the concentration of compounds, a calibration curve was made with high purity standards: p-hydroxybenzoic acid, vanillic acid, 3,4 dimethoxyphenol, gallic acid, chlorogenic acid, quercetin 3-rutinoside, and quercetin. Each sample was injected in triplicate.

### 5.8. Statistical Analysis

All experiments were established according to a completely randomized experimental design. The numerical variables in the in vitro and substrate assays were represented by their mean and standard deviation. In the in vitro assay, the numerical variables HL and RL were compared using the Student’s *t* test for independent groups. Differences between germination percentages (in vitro assay) were determined through the chi-square test (Fisher’s exact test). Dynamics of germination, plant and root length, and number of leaves were analyzed through a one-way ANOVA and the Tukey test for post hoc comparisons. A two-way ANOVA (Kruskal–Wallis test) was applied, and contrasts were performed to analyze the influence of the substrate (invaded and native) and the type of irrigation (water and aqueous extract of *T. monspessulana*) on the PL, NTL, and LMR. Comparison of the compound concentrations detected by HPLC was performed using a one-way ANOVA or Student’s *t* test. The assumption of normality and homogeneity of variances was verified using the Shapiro–Wilk and Levene’s tests, respectively. The results were analyzed with the SPSS 24.0 software and a significance level of 0.05 was used.

## Figures and Tables

**Table 1:** Statistical results (two-way ANOVA) for morphometric variables: plant length (PL), number of true leaves (NTL), and length of the main root (LMR) of *Nothofagus obliqua* seedlings, related to effects of substrate (native and invaded) and irrigation (water and aqueous extract of *Teline monspessulana*). Data are represented by mean and standard deviation. For each treatment, 10 seedlings were considered (n = 10). Different letters mean significant differences for *p* ≤ 0.05.

		Morphometric Variables		
		PL (cm)	NTL	LMR (cm)
Model	Mean	3.59 ± 0.15	4.55 ± 0.19	3.45 ± 0.16
	Statistician	50.2	54.3	48.8
	*p* value	<0.0001	<0.0001	<0.0001
Substrate	Invaded	2.89 ± 0.18 ^b^	3.70 ± 0.21 ^b^	2.79 ± 0.19 ^b^
	Native	4.30 ± 0.20 ^a^	5.40 ± 0.26 ^a^	4.11 ± 0.22 ^a^
	Statistician	59.88	21.24	46.67
	*p* value	<0.0001	<0.0001	<0.0001
Irrigation	Water	4.45 ± 0.20 ^a^	5.70 ± 0.26 ^a^	4.40 ± 0.18 ^a^
	Extract	2.73 ± 0.14 ^b^	3.40 ± 0.13 ^b^	2.49 ± 0.16 ^b^
	Statistician	88.48	35.29	98.93
	*p* value	<0.0001	<0.0001	<0.0001
	I-W	3.61 ± 0.24	4.55 ± 0.29	3.67 ± 0.18
	I-E	2.16 ± 0.13	2.85 ± 0.17	1.91 ± 0.17
Interaction	N-W	5.30 ± 0.17	6.85 ± 0.22	5.14 ± 0.22
	N-E	3.31 ± 0.17	3.95 ± 0.11	3.08 ± 0.20
	Statistician	2.18	0.23	0.70
	*p* value	0.1447	0.6322	0.4049

**Table 2:** Chemical composition of the native and invaded substrates.

Elements	Unit of Measurement	Native Value	Content Level (*)	Invaded Value	Content Level (*)
pH in water		6.24	Medium	6.03	Medium
Organic material	%	8.57	High	8.15	High
Nitrates (N-NO_3_)	mg kg^−1^	65.60	Medium	45.49	Medium
Ammonium (N-NH_4_)	mg kg^−1^	7.40	Low	7.40	Low
N available	mg kg^−1^	73.00	High	53.30	High
Olsen phosphorus	mg kg^−1^	19.10	Medium	3.50	Low
K available	mg kg^−1^	227.60	High	135.8	Medium
K interchangeable	cmol kg^−1^	0.58	High	0.35	Medium
Ca interchangeable	cmol kg^−1^	9.82	High	11.16	High
Mg interchangeable	cmol kg^−1^	3.03	High	1.69	High

* The level of element contents was established by [32].

**Table 3:** Quinolizidine alkaloids identified in aerial organs of *Teline monspessulana* by GC-MS. Legend: (-) not detected.

Compounds Name	Molecular Formula	Molecular Weight (g mol^−1^)	RA (%)			
			Leaves	Flowers	Stems	Pods
Caulophylline	C_15_H_20_N_2_O	204.27	2.19	16.10	-	-
Lupanine	C_15_H_24_N_2_O	248.36	-	12.20	-	-
Aphylline	C_15_H_24_N_2_O	248.36	3.97	20.30	27.70	20.81
Anagyrine	C_15_H_20_N_2_O	244.33	3.00	4.59	-	7.53
Sophocarpine	C_15_H_22_N_2_O	246.35	0.73	0.14	-	-
Ellipticine	C_17_H_14_N_2_	246.313	1.36	-	-	-
Cytisine	C_11_H_14_N_2_O	190.246	0.60	-	-	-

RA: Relative peak area (peak area relative to total peak area per plant organ).

**Table 4:** Profile and concentration of phenolic compounds of *Teline monspessulana* aerial organs detected through high-performance liquid chromatography (HPLC). Data are represented by mean and standard deviation. Different letters in the same row indicate significant differences for *p* ≤ 0.005 after a one-way ANOVA or Student’s *t* test. Legend: (-) not detected.

Compounds	Phenolic Concentrations (mg mL^−1^)				*p* Value
	Leaves	Flowers	Stems	Pods	
14-hydroxy benzoic	3.9 ± 0.06 ^c^	6.64 ± 0.07 ^b^	58.07 ± 0.05 ^c^	-	<0.001
Ellagic acid	-	4.09 ± 0.01	-		
Gallic acid	0.28 ± 4 × 10^−3 c^	0.44 ± 0.02 ^b^	1.48 ± 2 × 10^−3 a^	0.47 ± 0.02 ^b^	<0.001
Acid 5-(hydroxy methyl)furfural	-	-	4.41 ± 0.10	-	
p-tyrosol	-	-	2.10 ± 0.22	-	
Catechin	0.15 ± 1.9 × 10^−3^	-	-		
Vanillic acid	5.7 ± 0.01 ^c^	6.49 ± 0.03 ^b^	7.74 ± 0.02 ^a^	6.53 ± 0.02 ^b^	<0.001
Epicatechin	-	0.37 ± 0.02	-	-	
3,4-dimetho-ybenzyl alcohol	-	-	9.28 ± 0.1	-	
Vanillin	1.56 ± 0.13 ^c^	3.39 ± 0.10 ^a^	1.82 ± 0.02 ^b^	-	<0.001
Pinocembrin	-	1.58 ± 0.03	-	-	
Chlorogenic acid	0.41 ± 0.02 ^c^	2.24 ± 0.03 ^a^	0.47 ± 0.01 ^b^	2.08 ± 0.01 ^a^	<0.001
Caffeic acid	0.08 ± 0.03 ^b^	0.36 ± 0.22 ^b^	0.88 ± 1 × 10^−3 a^	-	<0.001
p-coumaric acid	0.17 ± 0.01 ^c^	6.97 ± 0.01 ^a^	0.26 ± 1 × 10^−4 b^	6.31 ± 0.01 ^a^	<0.001
Trans-ferulic	0.35 ± 0.01 ^b^	2.02 ± 0.01 ^a^	-	1.98 ± 0.01 ^a^	<0.001
Acid apigenin	4.64 ± 0.01	-	-	-	
Quercetin 3-rutinoside	1.9 ± 0.03	9.77 ± 5 × 10^−2^	-	-	<0.001
Quercetin 3- glucoside	5.01 ± 0.71 ^b^	31.11 ± 6 × 10^−3 a^	1.64 ± 1 × 10^−4 c^	-	<0.001
Myricetin	1.30 ± 0.01	-	-		
Quercetin	0.14 ± 4 × 10^−3 b^	0.14 ± 2 × 10^−3 b^	0.24 ± 1 × 10^−3 a^	-	<0.001
Kaempferol	2.82 ± 7 × 10^−3 a^	1.05 ± 4 × 10^−3 b^	0.34 ± 6 × 10^−3 c^	-	<0.001

## Data Availability

Data available on request, due to restrictions.

## Supplementary Materials

The following supporting information can be downloaded at: https://www.mdpi.com/article/10.3390/plants12193419/s1, Figure S1: Chromatogram from GC-MS (*Teline monspessulana* leaves); Figure S2: Chromatogram from GC-MS (*Teline monspessulana* pods); Figure S3: Chromatogram from GC-MS *(Teline monspessulana* stems); Figure S4: Chromatogram from GC-MS *(Teline monspessulana* flowers).
