# Supplementary material for: Teline monspessulana Can Harm the Chilean Native Tree Nothofagus obliqua: Effects on Germination and Initial Growth
Source: Plants (Basel). 2023 Sep 28;12(19):3419. doi: 10.3390/plants12193419 (PMC10575075; doi:10.3390/plants12193419)

Supplementary Figures

Figure S1. Chromatogram from GC-MS (*Teline monspessulana* leaves)

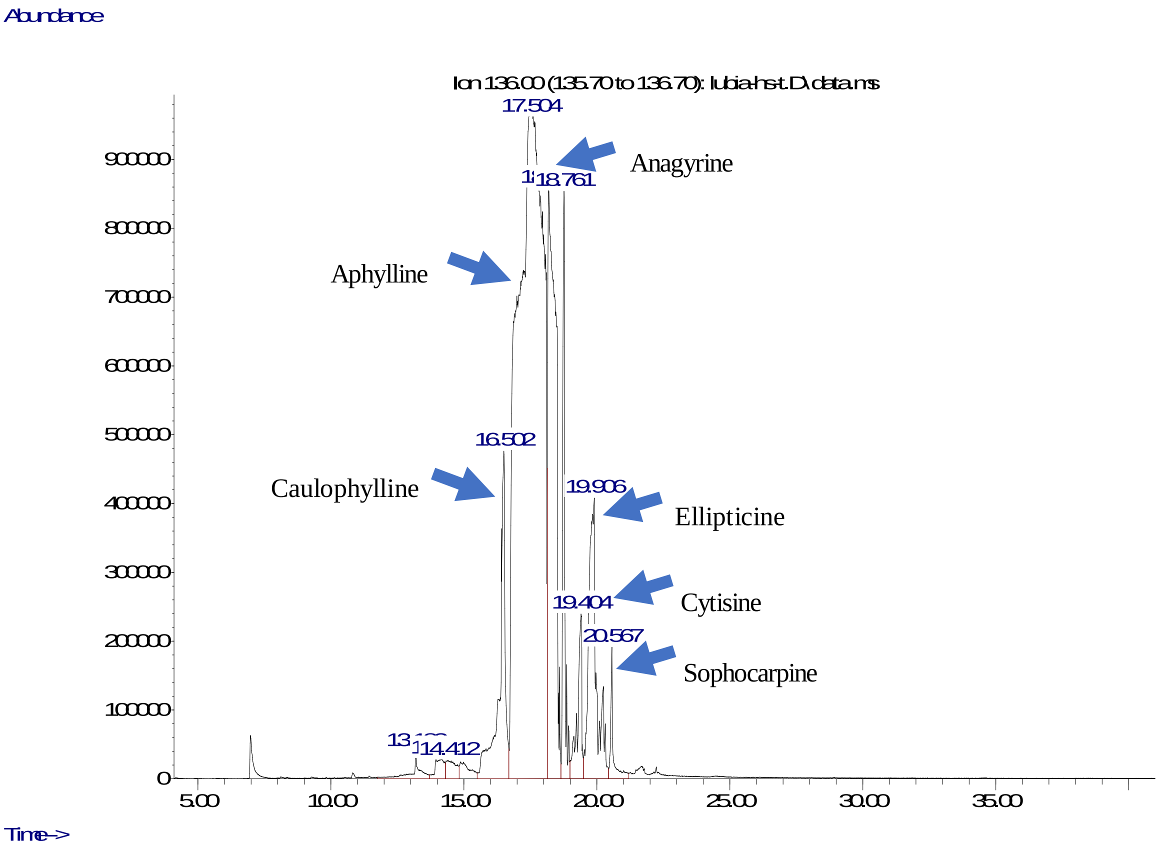

Figure S2. Chromatogram from GC-MS (*Teline monspessulana* pods)

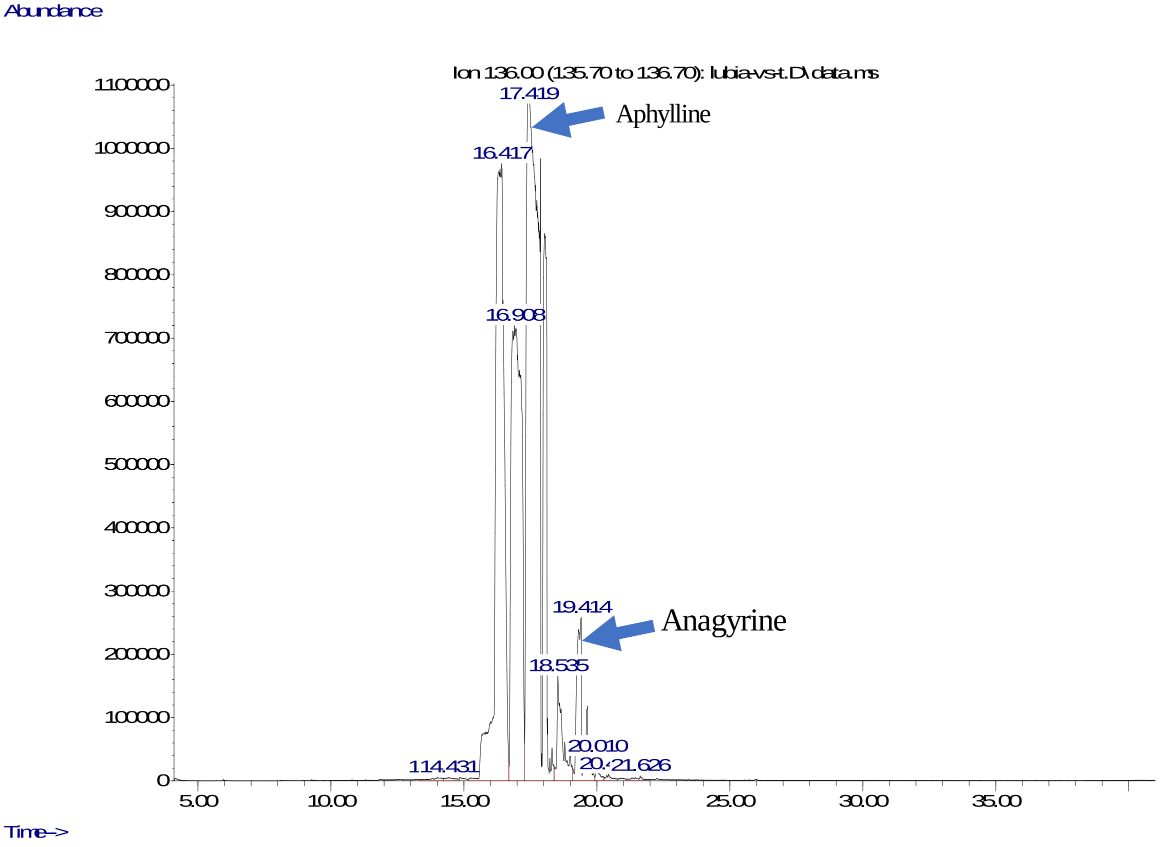

Figure S3 Chromatogram from GC-MS (*Teline monspessulana* stems)

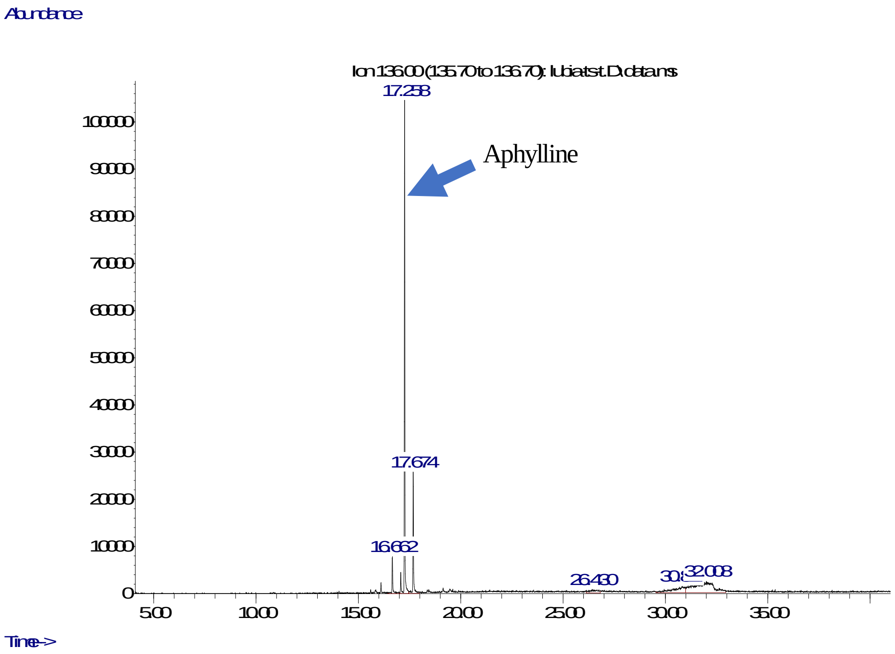

Figure S4. Chromatogram from GC-MS (*Teline monspessulana* flowers)

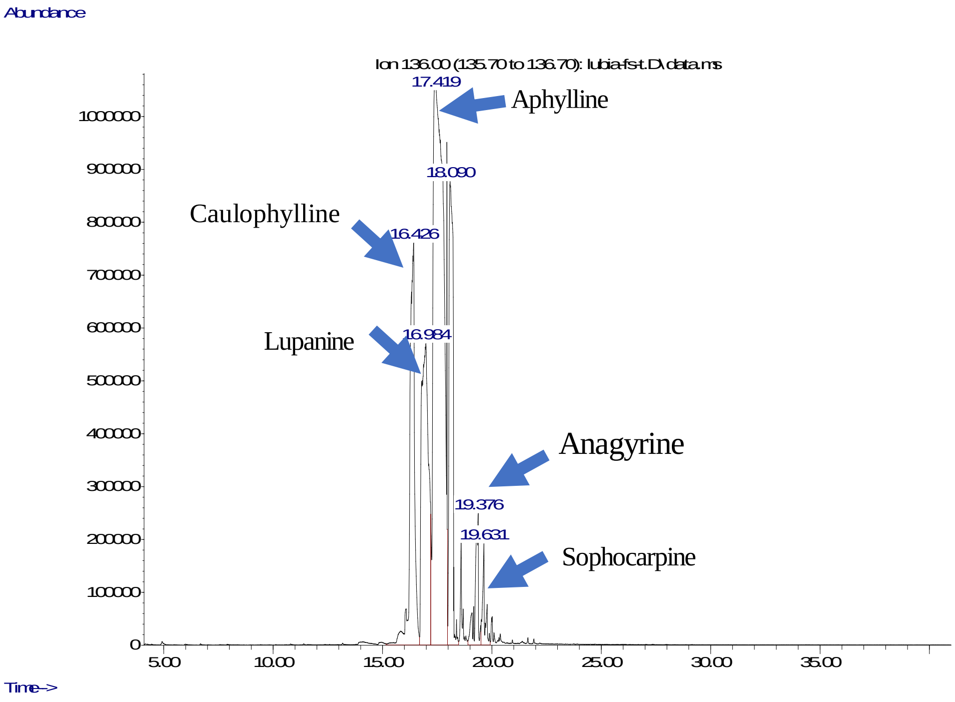

Supplement: Supplementary file 1 [file plants-12-03419-s001.zip › plants-2631441-supplementary.pdf]
